# Probe-Atom Distributions Obtained from Mixed-Solvent Molecular Dynamics Improve the Scoring of Docking Calculations

**DOI:** 10.1021/acs.jcim.6c00947

**Published:** 2026-08-31

**Authors:** Kaho Akaki, Keisuke Yanagisawa, Yutaka Akiyama

**Affiliations:** † Department of Computer Science, School of Computing, 13290 Institute of Science Tokyo, Meguro-ku, Tokyo 152-8550, Japan; ‡ Middle Molecule IT-based Drug Discovery Laboratory, Institute of Science Tokyo, Meguro-ku, Tokyo 152-8550, Japan

## Abstract

Structure-based virtual screening (VS) is widely used for the computational selection of drug candidates from compound libraries. Protein–ligand docking calculations are often performed as key steps in the early stages of this process. However, current docking calculations have limited accuracy. Thus, improvements are needed to more efficiently identify promising drug candidates. In this study, we performed mixed-solvent molecular dynamics (MSMD) simulations using four types of probe molecules to improve the accuracy of large-scale VS. We proposed a method for the modification of the docking scoring function for five selected atom classifications (XS_types). This approach integrated the grid free energy derived from the relevant atoms across the probe molecules. VS experiments conducted on nine target proteins showed improved accuracy, with the average EF_1%_ increasing from 6.65 to 7.36. Our method may facilitate drug discovery with higher accuracy than that of conventional methods.

## Graphical abstract

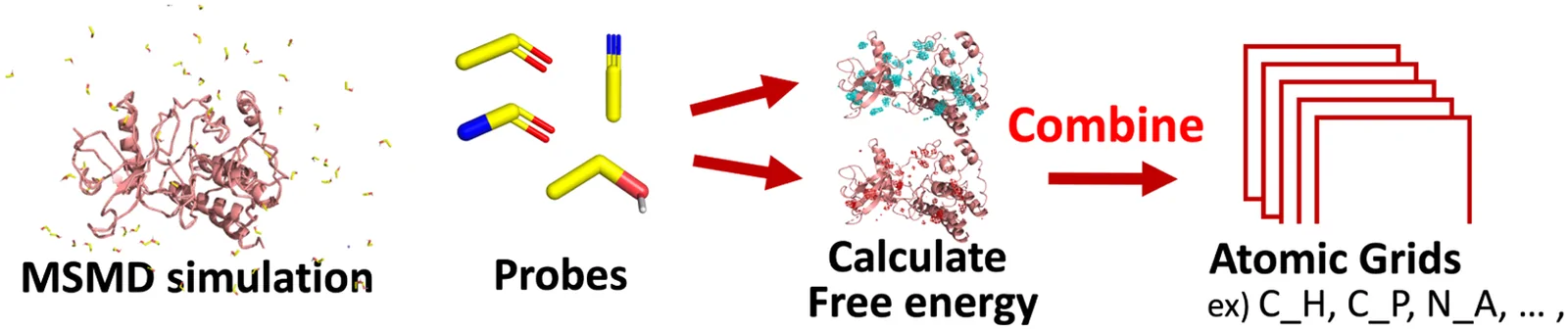

## Introduction

The drug discovery process requires an estimated average of 10–15 years and costs approximately 800 million to 1.8 billion US dollars.^1–3^ In addition, the total number of organic compounds synthesized ranges from 10^30^ to 10^60^.^4,5^ Therefore, methods for efficiently narrowing down candidate compounds are required. Virtual screening (VS) is a computational process for selecting candidate drug compounds from numerous compounds.^6,7^

Protein–ligand docking calculations (hereafter referred to as docking calculations) are widely used in the early stages of VS. Docking calculations predict the binding poses and affinities of compounds to target proteins.^8^ Numerous docking tools are available, including commercial tools, such as Glide^9^ and FlexX,^10^ and open-source tools such as AutoDock4,^11^ AutoDock Vina,^12^ REstretto,^13^ and rDock.^14^ The scoring function in docking calculations significantly affects prediction accuracy. For example, AutoDock Vina employs a scoring function that incorporates steric, hydrophobic, and hydrogen bond interactions between all heavy atoms of the protein and ligand. The scoring function is based on XS_type,^15^ which combines atomic identity with chemical properties such as hydrogen bond donor/acceptor capabilities, aromaticity, and hydrophobicity. In docking calculations for a single protein conformation, an atomic grid precalculates and stores scores to place atoms in each grid^16^ for faster calculation. Docking calculations efficiently predict binding poses using the above-mentioned methods. However, the scoring function does not explicitly account for solvent molecules, and atomic grids are generated from fixed protein structures.

Molecular dynamics (MD) simulations capture the dynamic behavior of molecules, such as proteins, ligands, and water molecules, at atomic resolution.^8^ Mixed-solvent MD (MSMD) simulations are performed by placing a protein in a box containing explicit water molecules and a limited number of small organic molecules (probes).^17^ The probes displace water from the protein surface and identify regions where drug-like organic molecules may bind favorably. The probability distribution of probe molecules from complete MSMD simulations has been utilized for binding site prediction,^18,19^ binding affinity prediction,^20–22^ pharmacophore construction,^23,24^ and cryptic site detection.^25–27^

Previous studies have used the grid free energy (GFE) obtained from MSMD to improve docking calculations.^20^ Arcon et al.^21,22^ developed AutoDock Bias, a modified version of AutoDock4, and proposed an approach that integrates MSMD into docking to enhance VS. Three independent MSMD simulations were performed using an ethanol concentration of 20% v/v (volume percentage concentration) for 20 ns. Frequently occupied solvent sites from the trajectories were converted into spherical bias terms and energetic restraints that favor specific protein–ligand interactions during docking. Compounds were docked and scored using this biased scoring function. However, this study had several limitations. Bias terms were applied to only a few spherical regions, as determined by the clustering algorithm results. Moreover, the clustering parameters required careful tuning based on expert knowledge. Additionally, only ethanol was used as a probe. This excluded carbonyl and carboxyl groups, which have distinct interactions, as well as nitrogen atoms, which are also important in protein–ligand interactions.

Ustach et al.^20^ predicted ligand-binding positions using the SILCS (Site Identification by Ligand Competitive Saturation) and SILCS-MC methods. They performed SILCS grand canonical Monte Carlo MD (GCMC-MD) on the simulated system, followed by 100 production cycles. Each cycle involved 200,000 GCMC steps, 5,000 steps of steepest descent minimization, 100 ps equilibration MD, and 1 ns production MD simulations. These simulations included a mixture of eight probe molecules at a concentration of 0.25 M. FragMaps, which correspond to GFEs, were then calculated from the obtained occupancy probability. The SILCS-MC method uses these FragMaps to estimate binding positions and affinities of the ligands. However, this method was designed to refine binding-pose predictions for known binders. Its ability to discriminate binders from nonbinders in a VS context has not been demonstrated. In this study, we propose a new integration method that combines MSMD with docking to improve the accuracy of large-scale VS. This method generates GFEs for five XS_types, an atom classification scheme used in AutoDock Vina, based on the molecular distribution of multiple probes from MSMD simulations, which are then incorporated into the docking scoring function, while preserving the original potential surface without simplifying it as a set of simple spherical regions.

## Materials and Methods

### Methods Overview

The workflow of the proposed method is illustrated in Figure 1.

(1) Multiple independent MSMD simulations were performed for each probe molecule.
(2) The probability distribution map (PMAP) of probe atoms from MSMD trajectories was identified, and the GFEs for five XS_types were calculated.
(3) Several original atomic grids were modified with GFEs, which were then used to perform docking.

**Figure 1:**
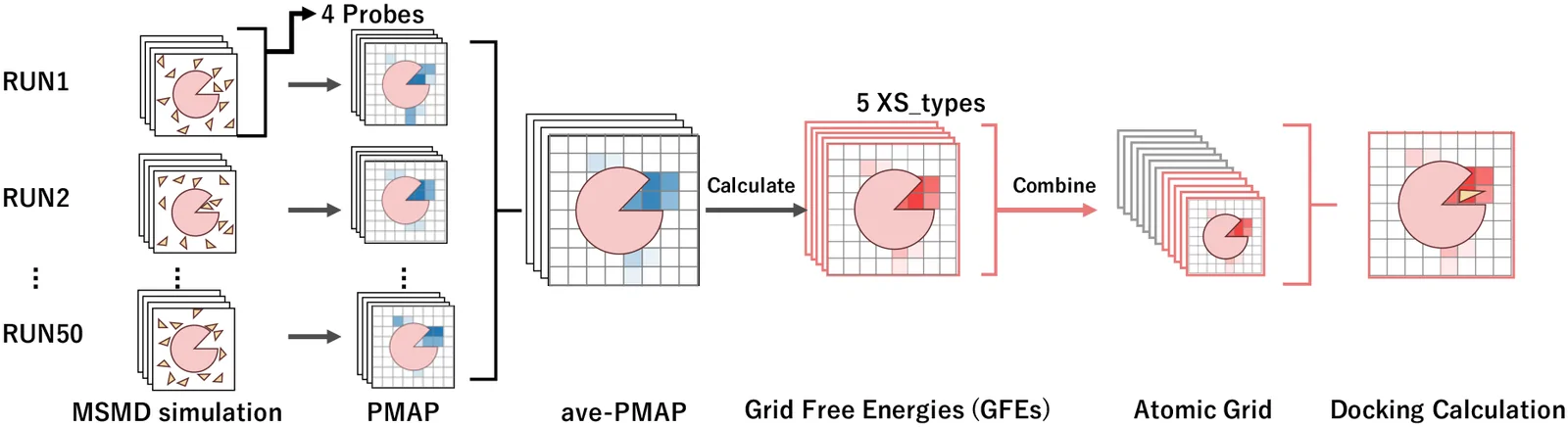
Workflow of the proposed method.

The following sections provide step-by-step descriptions of methodological details.

### Preparations for MSMD Simulation

#### Protein Preparation

The Protein Preparation Wizard and Prime^28^ in the Schrödinger suite 2018-3 (Schrödinger, Inc., New York, NY, USA) were used to fill the missing loops, side chains, and atoms. Ligands and cofactor molecules were then removed. The N- and C-termini were capped with *N*-methyl amide and acetyl groups, respectively. Hydrogen atoms were placed considering the hydrogen bonding and ionization states at pH = 7 with PROPKA.^29^ Water molecules with fewer than two hydrogen bonds to a protein in the crystal structure were removed, followed by structural optimization.

#### Probe Preparation

The GFEs were applied to the terms of the scoring function corresponding to the various XS_types. XS_type refers to the classification of the atomic grids used for scoring; this terminology was introduced by AutoDock Vina.^12^

To determine which XS_types are effective in VS experiments, we analyzed the occurrence frequency of XS_types for all active and decoy compounds in the DUD-E 102 targets^30^ totaling 1,434,100 compounds. Table 1 lists the frequencies of each XS_type. The total number of atoms corresponding to the XS_types in the entire DUD-E 102 target data set was 39,279,444. The most frequent was C_H, which represented hydrophobic carbon atoms with 17,168,313 occurrences (43.7%), followed by C_P, which represented polar carbon atoms with 11,367,252 occurrences (28.9%).

**Table 1:** Occurrence Frequency of XS_types in All Compounds within the DUD-E 102-Target Data Set^a^

XS_type	Occurrence	Proportion (%)
C_H (hydrophobic carbon)	17,168,313	43.7
**C_P (polar carbon)**	**11,367,252**	**28.9**
**O_A (H-bond acceptor oxygen)**	**3,369,146**	**8.6**
**N_D (H-bond donor nitrogen)**	**1,966,109**	**5.0**
**N_A (H-bond acceptor nitrogen)**	**1,489,764**	**3.8**
N_P (polar nitrogen)	1,025,446	2.6
S_P (polar sulfur)	901,322	2.3
O_P (polar oxygen)	604,313	1.5
F_H (hydrophobic fluorine)	395,700	1.0
Cl_H (hydrophobic chlorine)	349,782	0.9
O_DA (H-bond donor/acceptor oxygen)	**336,693**	**0.9**
Br_H (hydrophobic bromine)	157,567	0.4
N_DA (H-bond donor/acceptor nitrogen)	129,022	0.3
I_H (hydrophobic iodine)	12,781	0.0
P_P (polar phosphorus)	6,217	0.0
OTHER (other)	9	0.0
O_D (H-bond donor oxygen)	8	0.0
Met_D (H-bond donor metal)	0	0.0
Total of all XS_types	39,279,444	100.0

a The five XS_types whose scores were modified in this study are shown in bold. Percentages may not sum to exactly 100.0% because values are rounded to one decimal place.

Based on the above results, we selected five XS_types for the atomic grid score modification: C_P (polar carbon atoms adjacent to nitrogen or oxygen), O_A (hydrogen bond acceptor oxygen atoms), N_D (hydrogen bond donor nitrogen atoms), N_A (hydrogen bond acceptor nitrogen atoms), and O_DA (hydrogen bond donor/acceptor oxygen atoms). These correspond to ranks 2 through 5 and O_DA, which is derived from the ethanol probe used by Arcon et al.^21^ The top-ranked C_H (hydrophobic carbon atoms) was excluded. The total occurrence frequency of the five selected XS_types (C_P, N_A, N_D, O_A, and O_DA) was 18,528,964, which corresponds to 47.2% of all XS_type atoms. In other words, the proposed method applied modified scoring with the GFE to approximately half of the atoms in the compound libraries of the DUD-E database.

As shown in Table 2, one or more probe molecules were used to modify the score of each XS_type. Conversely, the statistics for multiple XS_types can be obtained from a single probe molecule. Four probe molecules were used: acetaldehyde, acetonitrile, formamide, and ethanol (Figure 2). For example, C_P was mapped to the atoms contained in acetaldehyde, acetonitrile, formamide, and ethanol. The XS_types N_A, N_D, O_A, and O_DA were mapped to atoms in acetonitrile, formamide, acetaldehyde/formamide, and ethanol, respectively, to specifically probe the hydrogen bonding and polar interactions. Imidazole was selected as the probe for N_A in the study by Ustach et al.^20^ However, imidazole contains both N_A and N_D, making it difficult to distinguish which contribution is responsible for the interaction. Additionally, because imidazole is a relatively large molecule with a ring structure, increased noise during sampling remains a possibility. Therefore, acetonitrile, which has a simpler structure, was selected as the probe for N_A in the present study. Furthermore, C_H (hydrophobic carbon atoms) have weaker interactions than hydrogen bonds, and the interactions at binding sites involving these atoms tend to be of low specificity. Therefore, its atomic grid score was not targeted for modification in this study.

**Table 2:**
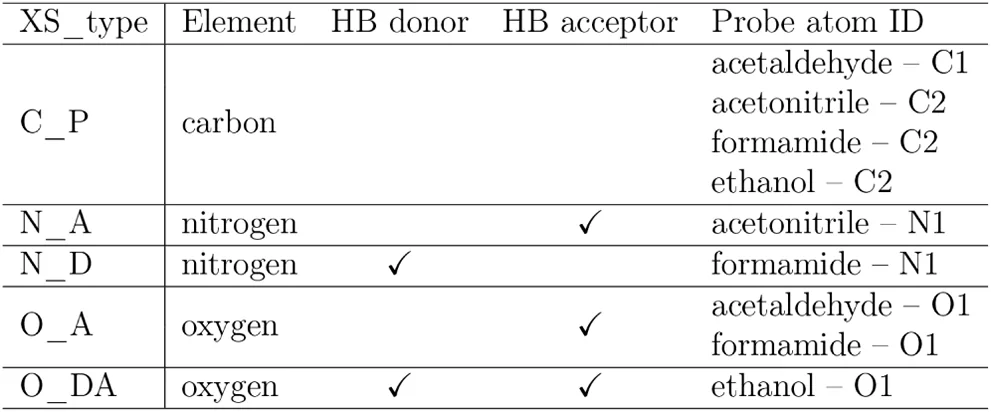
Assignments of Probe Molecules to XS_types Whose Scores Were Modified in This Study^a^

a The five selected XS_types represent key pharmacophoric features involved in protein–ligand interactions, especially hydrogen bonding and polar contacts. The atom ID corresponds to Figure 2.

**Figure 2:**
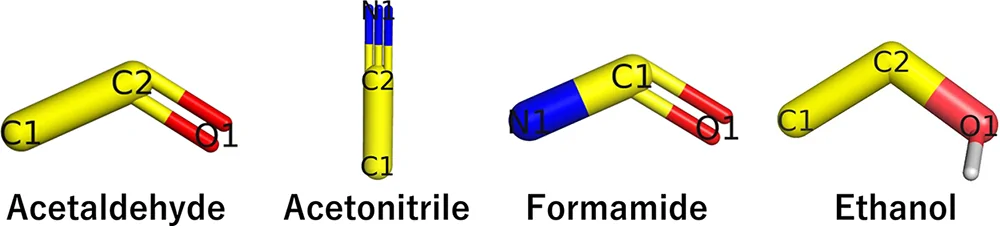
Probe molecules. Only heavy and polar hydrogen atoms are depicted, with IDs specified for each atom.

The restrained electrostatic potential procedure in the Antechamber module of AmberTools was employed to fit partial charges to the electrostatic potential, which was calculated using Gaussian 16 Rev B.01.^31^ First, all probe structures were optimized at the B3LYP/6-31G­(d) level. The electrostatic potentials were then calculated at the HF level using the optimized structures. The centers of the electrostatic potentials were located at the center of each atom. Additional force field parameters for the probes were derived using the general Amber force field 2 (GAFF2).

### MSMD Simulation Protocol

We conducted MSMD using the EXPRORER protocol.^32^ The initial positions of the probes affect the results, especially in short MD simulations. This initial position dependency affects GFE generation. To achieve efficient sampling, the following protocol was performed independently 50 times with different initial probe coordinates. The procedure was divided into three steps as described below:

### Initial System Generation

Probe molecules were randomly placed around the protein at a concentration of 1.0  M using PACKMOL 18.169.^33^ Next, the system was solvated by adding water molecules to the LEaP module of AmberTools. The Amber ff14SB^100^ force field and TIP3P model^34^ were used for the protein and water molecules, respectively. Additionally, a Lennard–Jones force field term with the parameters (ϵ = 10^–6^ kcal/mol; *R*min = 20 Å) was introduced only between the centers of the probe molecules to prevent their aggregation.

### Minimization, Heating, and Equilibration

After the construction of the initial structures, the systems were minimized for 200 steps using the steepest descent algorithm with harmonic position restraints on the heavy solute atoms (force constant, 10 kcal/mol/Å^2^) to prevent deviations from the initial structures. These were subsequently minimized by a further 200 steps using the steepest descent algorithm without positional restraints. After minimization, the system was gradually heated to 300 K during 200 ps constant-*NVT* MD simulations with harmonic position restraints on the solute heavy atoms (force constant, 10 kcal/mol/Å^2^). Owing to the high concentration of probe molecules, strong intermolecular repulsions occasionally caused system instability, which was detected as a simulation failure during the heating phase. In such cases, heating was restarted for an extended duration of 1,000 ps under the same restraint conditions to ensure system stability. Equilibration was subsequently performed by decreasing the restraint force constants. During the subsequent 800 ps constant-*NPT* MD simulations at 300 K and 10^5^ Pa, the force constants of the position restraints were gradually reduced from 10 kcal/mol/Å^2^ to 0 kcal/mol/Å^2^. The P-LINCS algorithm^35^ was used to constrain all bond lengths involving hydrogen atoms, which allowed the use of 2 fs time steps. The temperature and pressure were controlled using a stochastic velocity rescaling algorithm,^36–38^ and a Berendsen barostat,^39^ respectively. The simulations were performed using GROMACS 2021.5.^40^ The ParmEd module^41^ was used to convert the AMBER parameter/topology file format to that used in GROMACS.

### Production Run

After equilibration, 150 ns constant-*NPT* MD simulations were performed at 300 K and 10^5^ Pa. Position restraints with a force constant of 0.01 kcal/mol/Å^2^ were applied to all heavy atoms of the protein. The parameters for the MSMD simulations were the same as those for the equilibration step, except for pressure control. The Parrinello–Rahman barostat^42^ was used instead of the Berendsen barostat. Snapshots were captured every 10 ps in the 100–150 ns. Thus, 5,000 snapshots were produced per MSMD simulation.

### Derivation of GFE

#### Generation of Spatial Probability Distribution Map

After the production runs were conducted, all trajectories were processed to generate a spatial probability distribution map (PMAP). For each probe, all heavy atoms in the snapshots were binned into 1 Å × 1 Å  × 1 Å grid voxels, with separate grids generated for each atom type; the voxel occupancy was counted for each grid. Because the binding site exists on the protein surface, only voxels within 5 Å of the protein atoms were treated. Each voxel count was divided by the number of snapshots to obtain occupancy probabilities. One PMAP corresponds to one trajectory. Therefore, 50 PMAPs were generated for each probe atom. The averaged PMAPs (ave-PMAPs) for each atom were constructed by averaging the corresponding voxel values in the PMAPs generated from each independent trajectory.

### Conversion to GFE

Boltzmann-based transformation enables the estimation of the free energy of each position, called GFE.^43^ GFE is defined as eq 1:

1 $${\mathrm{GFE}}_{x,y,z}^{a}=-RT\ln\left(\frac{{\mathrm{occupancy}}_{x,y,z}^{a}}{\left\langle {\mathrm{bulk occupancy}}^{a}\right\rangle }\right)$$

*R* is the gas constant (1.99 × 10^–3^ kcal/mol/K), and *T* is the temperature set to 300 K. ${\mathrm{occupancy}}_{x,y,z}^{a}$ represents the probability that a probe atom is present within a volume of 1 Å^3^ centered at the coordinates *x*, *y*, and *z*, whereas bulk occupancy denotes the average probability per 1 Å^3^ volume when the probe molecules are uniformly distributed in space. The GFE was derived by calculating the ratio of the local probability of the presence of the probe atom to the corresponding bulk average probability and subsequently converting this ratio into an energy quantity.

Because the simulation system contains not only probe molecules but also protein and water molecules, only the atoms belonging to the probe molecules were considered. The GFE was derived from the probability that an atom exists within the entire probe molecule. The use of a molecular probe rather than a single atom enables the incorporation of interactions that atomic grids cannot capture. On the other hand, the calculated energy reflects not only the interactions of the target atom but also those of neighboring atoms within the probe molecule. This incorporation of multiatom effects resulted in more pronounced variations in the probability distribution than observed in single-atom calculations. Additionally, the concentration of probe molecules used in the simulations is substantially higher than that typically present under ligand-binding conditions, which may lead to an overestimation of the binding free-energy scores. As a result, the GFE values obtained from MSMD were sometimes < −2.0 kcal/mol. This is markedly stronger than the range produced by the original scoring function, whose values for individual atoms are typically no lower than approximately −1.0 kcal/mol. To correct for this substantial discrepancy in score ranges, we scaled the GFE values to match the mean and standard deviation of the atomic grid values. In this process, the scaling parameters (mean and standard deviation) were derived only from favorable values (less than 0 kcal/mol), whereas values equal to or greater than 0 kcal/mol remained unchanged. The scaled GFE is hereafter referred to as the GFE.

### Docking Calculations with GFEs

Two steps of conversions were required to incorporate the GFE obtained from MSMD into the docking calculation as atomic grid values: (1) spatial alignment of the GFE grid to match the center coordinates, size, and grid spacing of the docking box, and (2) mapping from the probe types used in MSMD to the XS_types used in the docking scoring function. For spatial alignment, the GFE grid was first trimmed to the region corresponding to the docking box, and the values were linearly interpolated to match the grid spacing used in the docking calculations. Some XS_types appear in multiple probes. For example, C_P is shared by acetaldehyde, acetonitrile, formamide, and ethanol, whereas O_A is shared by acetaldehyde and formamide. For XS_types appearing across multiple probes, the minimum of their respective values was employed. Subsequently, for each voxel, the minimum of the MSMD-based GFE and Vina scores was obtained for each XS_type. This approach was designed to leverage the strengths of both MSMD and the Vina scoring function to capture the favorable interactions identified by either method.

### Target Proteins and Compound Libraries

We used the nine target proteins that Arcon et al.^21^ employed in the VS experiments. The PDB IDs and descriptions of each target protein are shown in Table 3.

**Table 3:** Data Set Used for VS Evaluation

Target	PDB ID	Description	Active Compounds	Decoy Compounds
AmpC	1L2S	AmpC β-lactamase	48	2,847
BRD4	4ZC9	Bromodomain-containing protein 4	249	15,048
CDK2	1H00	Cyclin-dependent kinase 2	474	27,831
DHFR	3NXO	Dihydrofolate reductase	231	17,170
FGFr1	3C4F	Fibroblast growth factor receptor 1	139	8,691
FXa	3KL6	Factor Xa	537	28,247
Gal-3	1KJR	Galectin-3	37	1,703
HIVpr	1XL2	HIV-1 protease	536	35,688
PDE5A	1UDT	Phosphodiesterase 5A	398	27,520

A compound library comprising active and decoy compounds specific to each target protein was constructed. Seven of the nine target proteins were obtained from the DUD-E database.^30^ The remaining two targets (BRD4 and Gal-3) were obtained from the data set published by Arcon et al.^21^ The DUD-E data set provides active ligands for each target protein, along with decoy molecules that share similar physicochemical properties but are structurally dissimilar to the active compounds, thereby enabling an unbiased evaluation of the VS performance. Preprocessing, including ionization-state generation, was performed on all compound libraries. The ionization states were estimated using LigPrep^44^ within the pH range 5–9.

### Protein–Compound Docking Calculations

Docking calculations were performed using AutoDock Vina with the default parameters. The docking search space was defined as a box enclosing the cocrystallized ligand extended by 5 Å beyond the outermost atoms of the ligand in both directions along each axis. The resulting box dimensions for each target protein are listed in Table 4.

**Table 4:** Box Information of Nine Target Proteins

Protein	Box center (Å)	Box size (Å^3^)
AmpC	(23.56, 5.74, 14.40)	18 × 14 × 18
BRD4	(−9.86, −5.61, 1.64)	18 × 16 × 18
CDK2	(1.97, 27.56, 8.82)	20 × 24 × 16
DHFR	(15.24, −5.84, −0.20)	18 × 16 × 20
FGFr1	(6.29, 4.96, 18.76)	16 × 14 × 20
FXa	(2.16, −8.65, −13.35)	20 × 20 × 22
Gal-3	(−0.90, −9.25, 0.55)	26 × 16 × 16
HIVpr	(20.14, −2.72, 18.30)	22 × 16 × 22
PDE5A	(1.44, 67.02, 83.53)	18 × 24 × 18

### Convergence Assessment of Probability Distribution Maps

To confirm that MSMD simulations yield stable and reproducible results, we assessed the convergence of the probe molecule distributions. Convergence was evaluated using the overlap coefficient (OC), proposed by Raman et al.^45^ The OC quantifies the overlap between two PMAPs on a scale from 0 to 1, where a value of 1 indicates identical distributions, and is defined as eq 2:

2 $$\mathrm{OC}=\overset{N}{\underset{i=1}{\sum }}\min\left(\frac{Q_{i}^{1}}{\overset{N}{\underset{j=1}{\sum }}Q_{j}^{1}},\frac{Q_{i}^{2}}{\overset{N}{\underset{j=1}{\sum }}Q_{j}^{2}}\right)$$

where *N* is the total number of voxels in the PMAP, and $Q_{i}^{1}$ and $Q_{i}^{2}$ are the occupancy counts of the *i*-th voxel in the two PMAPs being compared. The OC is obtained by first normalizing each voxel occupancy count by the sum of all voxel occupancy counts within the corresponding PMAP and then summing the smaller of the two normalized values across all voxels. Raman et al. reported that the OC does not behave linearly; relatively small differences between two distributions can reduce the value from 1.0 to approximately 0.8, and values exceeding 0.6 are considered indicative of sufficient similarity between PMAPs. In the present study, for each probe, 50 trajectories were divided into two groups (Group 1: trajectories 1–25; Group 2: trajectories 26–50), and the mean probability density for each group was computed. The OC between the two groups was then calculated over a 20 Å × 20 Å × 20 Å subregion centered on the binding pocket.

### Evaluation Experiments Based on VS Performance

VS is a computational approach for efficient identification of drug candidates from large compound libraries. In this study, we evaluated both accuracy and computation time, with a primary focus on VS accuracy. Subsequently, we investigated whether the proposed modifications to the scoring function improved VS accuracy. The experiments were performed on nine target proteins, and the numbers of active and decoy compounds for each target protein are shown in Table 3.

We evaluated VS accuracy using two metrics: the enrichment factor (EF) and the area under the receiver operating characteristic curve (ROC-AUC). In the VS, all compounds were ranked by docking score, and this ranking was subsequently used to assess screening performance. The EF is defined as eq 3:

3 $${\mathrm{EF}}_{x\%}=\frac{{\mathrm{Pos}}_{x\%}/{\mathrm{All}}_{x\%}}{{\mathrm{Pos}}_{100\%}/{\mathrm{All}}_{100\%}}$$

where Pos_*x*%_, All_*x*%_, Pos_100%_, and All_100%_ represent the number of active compounds in the top *x*% of the screened compounds, number of compounds in the top *x*% of the screened compounds, total number of active compounds, and total number of compounds, respectively. EF_x%_ = 1.0 corresponds to random selection performance. In this study, we calculated EF_1%_ and EF_10%_. The ROC curve was plotted with the true-positive rate on the vertical axis and the false-positive rate on the horizontal axis, generated by varying the docking score threshold. An ROC-AUC greater than 0.5 indicates that the ROC curve lies above the diagonal, whereas random predictions yield an ROC-AUC of 0.5. We also evaluated the execution time (CPU core seconds). The computation time was measured only once.

### Computing Environment

The experiments were conducted using TSUBAME4.0, a supercomputer at the Institute of Science Tokyo. Each computing node had two AMD EPYC 9654 CPUs (2.4 GHz, 96 cores each) and four NVIDIA H100 GPUs.

To accelerate the MSMD simulations, we executed five simulations concurrently per GPU. This was achieved using the NVIDIA Multi-Process Service (MPS), which allows multiple CUDA processes to efficiently share a single GPU.^46^ MPS significantly improves GPU core utilization by enabling the concurrent execution of multiple MSMD processes on the same device. As a result, approximately 200 node hours were required to obtain the GFEs of the five XS_types for one protein (four probes, 50 simulations of 150 ns each).

## Results

### Generation of Spatial Probability Distribution Map

The convergence of the probe molecule distributions obtained from the MSMD simulations was evaluated using the overlap coefficient (OC). The resulting OC values were approximately 0.95 across all systems, with only minor variation among proteins, confirming a high degree of convergence (Table 5). These values are substantially higher than the OC values reported by Raman et al. The probability density distributions of the acetonitrile carbon atom for BRD4, which exhibited the highest OC of 0.984, and for PDE5A, which exhibited the lowest OC of 0.933, are shown in Figure 3a and 3b, respectively. Both distributions show agreement not only in the locations of high-scoring regions but also in their spatial extent, which are almost identical. These results suggest that approximately 50 independent 150 ns simulations are sufficient to obtain stable, converged MSMD results.

**Figure 3:**
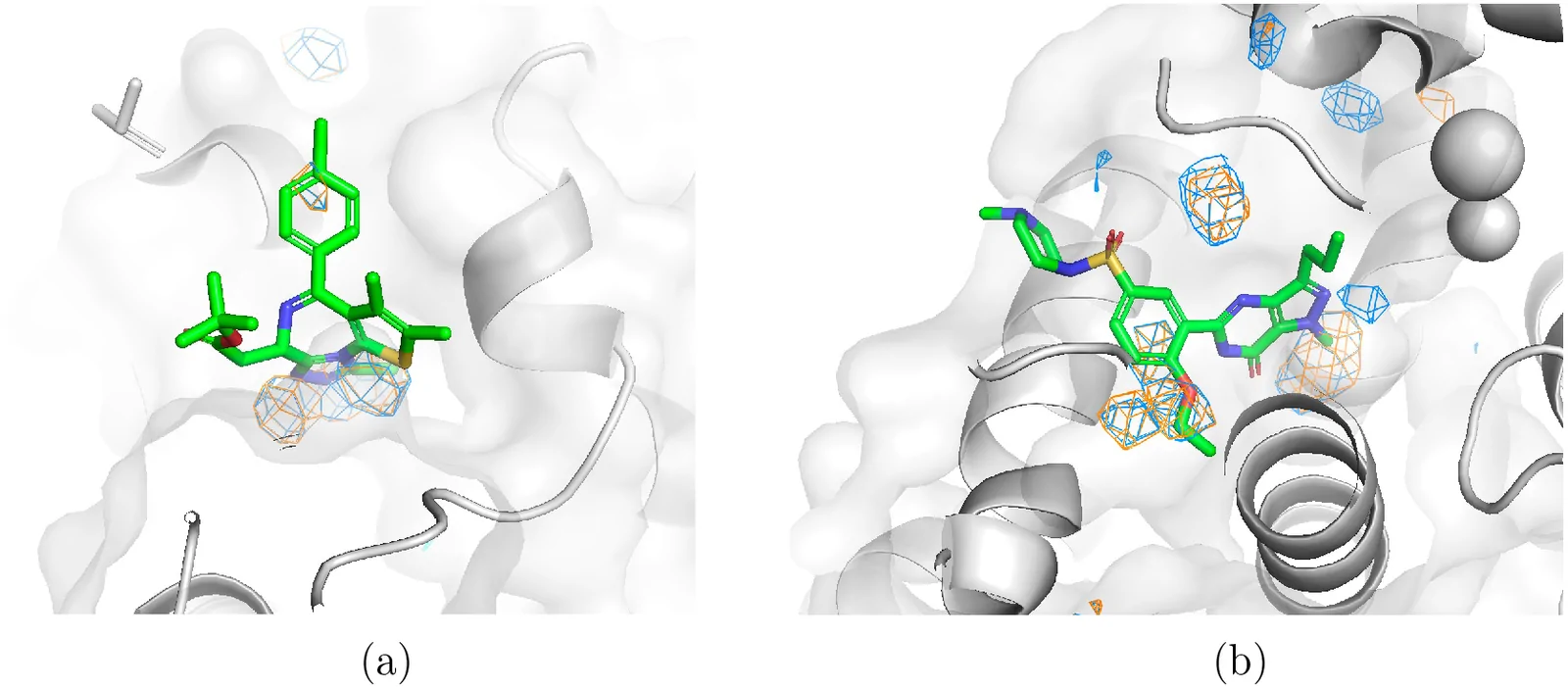
Convergence of the probability distribution obtained from MSMD. (a) The cocrystallized structure of the BRD4 protein (gray) and the ligand (green) (PDB ID: 3MXF), together with the probability distribution for the nitrile carbon atom of acetonitrile; (b) The cocrystallized structure of the PDE5A protein (gray) and ligand (green) (PDB ID: 1UDT), together with the corresponding probability distribution. The probability distributions, divided into two groups, are shown in orange and blue.

**Table 5:** Overlap Coefficient Calculated for Nine Target Proteins and Four Types of Probe Molecules

	Acetaldehyde		Acetonitrile		Formamide			Ethanol	
Protein	C2	O1	C2	N1	C1	N1	O1	C2	O1
AmpC	0.977	0.976	0.975	0.975	0.966	0.968	0.966	0.974	0.975
BRD4	0.983	0.982	0.984	0.983	0.978	0.978	0.976	0.981	0.980
CDK2	0.948	0.949	0.940	0.943	0.940	0.945	0.940	0.937	0.938
DHFR	0.956	0.958	0.968	0.969	0.978	0.978	0.979	0.980	0.981
FGFr1	0.963	0.967	0.961	0.963	0.943	0.945	0.945	0.945	0.947
FXa	0.944	0.945	0.946	0.949	0.951	0.953	0.952	0.945	0.945
Gal-3	0.956	0.956	0.951	0.954	0.942	0.943	0.941	0.942	0.944
HIVpr	0.971	0.973	0.963	0.965	0.960	0.963	0.961	0.957	0.960
PDE5A	0.951	0.953	0.933	0.937	0.953	0.954	0.952	0.954	0.955

### Shape Characteristics of GFE Obtained from MSMD

As an example of the GFE shapes obtained from the MSMD simulations, Figure 4 shows the GFE for the polar carbon atoms (C_P) in FGFr1 and CDK2. Some shapes resembled continuous ellipsoids, as highlighted by red circles. However, simple spheres cannot adequately represent these shapes. Therefore, the proposed method is considered more appropriate than that proposed by Arcon et al.

**Figure 4:**
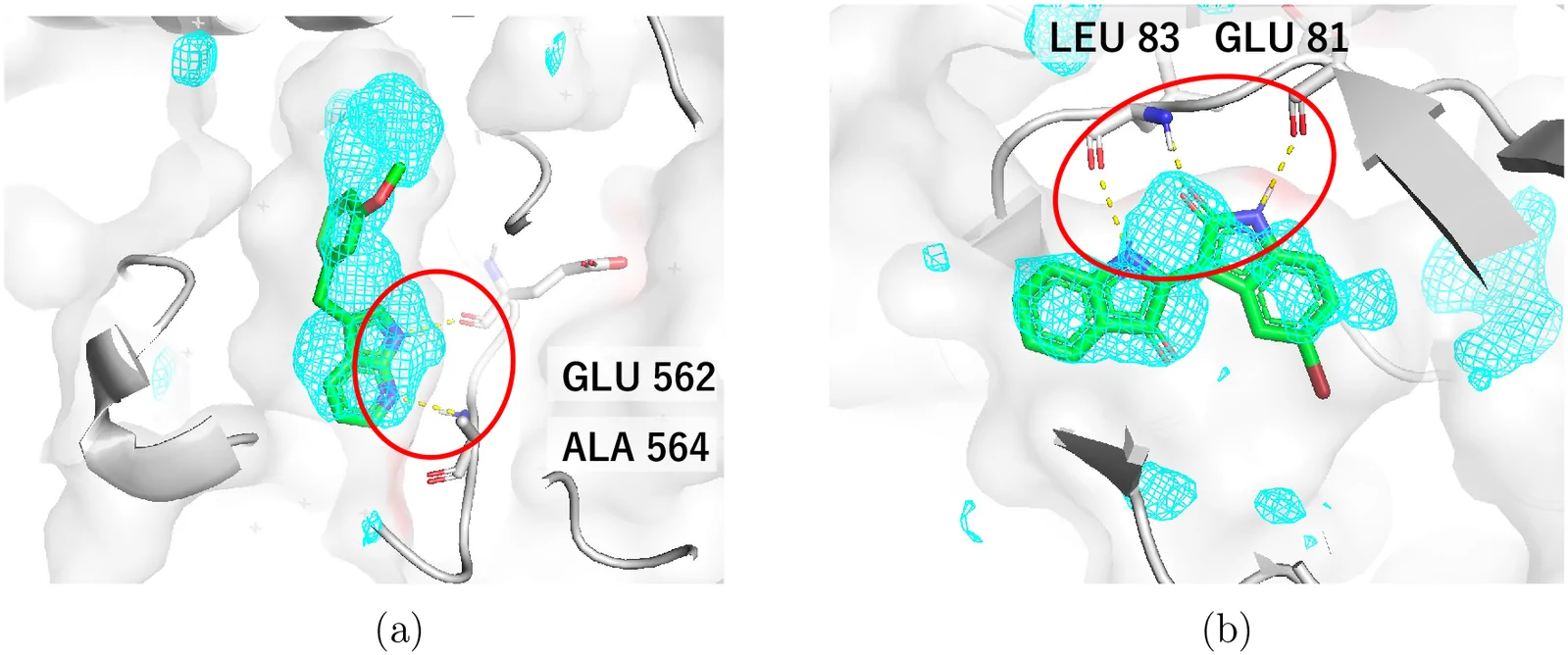
GFE shapes obtained from MSMD. (a) The FGFr1 protein structure (gray) and binding pose of an inhibitor (green, PDB ID: 3C4F). (b) The CDK2 protein structure (gray) and binding pose of an inhibitor (green, PDB ID: 2BHE). The free energy distributions represent the polar carbon atoms (cyan). Dashed yellow lines indicate hydrogen bonds.

### VS Accuracy

The VS accuracies are presented in Table 6. First, EF_1%_ improved for five targets but decreased for three, with average value increased from 6.65 to 7.36. The CDK2 target exhibited the largest improvement, with an increase in EF_1%_ of more than 5. The ROC-AUC values are listed in Table 6. The ROC curves are displayed in Supporting Information Figures  S1–S 9. Similarly, the average value increased from 0.659 to 0.671 for the ROC-AUC.

**Table 6:** VS Accuracy for Target Proteins^a^

	EF_1%_		ROC-AUC	
Target	w/o GFEs	w/ GFEs	w/o GFEs	w/ GFEs
AmpC	**6.26**	1.29	**0.544**	0.485
BRD4	1.32	**3.70**	0.648	**0.674**
CDK2	8.61	**13.78**	0.731	**0.738**
DHFR	1.11	**1.82**	**0.716**	0.702
FGFr1	12.07	**15.07**	0.616	**0.722**
FXa	**13.32**	12.27	0.804	**0.809**
Gal-3	0.00	0.00	0.532	**0.544**
HIVpr	**3.53**	2.65	**0.701**	0.660
PDE5A	13.65	**15.68**	0.643	**0.709**
Average	6.65	**7.36**	0.659	**0.671**

a Values with better accuracy are shown in bold.

### Execution Time of Docking

The execution times per compound for each target are shown in Table 7, which compares the docking calculations performed with and without GFEs. The execution times remained largely comparable between the two conditions. This suggests that GFE-based scoring did not substantially increase the computational cost of docking and did not adversely affect the complexity of the pose search.

**Table 7:** Execution Times Per Compound with Active and Decoy Compounds (CPU Core Seconds)^a^

Target	Without GFEs		With GFEs	
AmpC	41.3 sec.		34.8 sec.	
BRD4	74.8 sec.	(66.5 sec.)	63.9 sec.	(63.2 sec.)
CDK2	71.3 sec.		59.1 sec.	
DHFR	75.7 sec.		67.9 sec.	
FGFr1	154.7 sec.		137.3 sec.	
FXa	145.6 sec.		126.5 sec.	
Gal-3	410.9 sec.	(215.1 sec.)	380.2 sec.	(221.7 sec.)
HIVpr	121.7 sec.		107.6 sec.	
PDE5A	91.0 sec.		76.7 sec.	
Average	131.9 sec.		117.1 sec.	

a For BRD4 and Gal-3, compounds with a molecular weight of 600 or greater, or with 20 or more rotatable bonds, were excluded in accordance with the DUD-E protocol.^30^ The values in parentheses indicate the computation time calculated using only the remaining compounds after this exclusion.

## Discussion

### GFE Correctly Detected Hydrogen Bonds

In the proposed method, we modified the atomic grids for five XS_types: C_P, O_A, N_D, N_A, and O_DA. To determine how the GFE captures hydrogen bonding interactions compared to the original docking scoring function, we examined the complex structure of CDK2, one of the target proteins that showed a substantial increase in EF_1%_, with its inhibitor, as shown in Figure 5. In CDK2, three hydrogen bonds were formed between the ligand and the residues GLU81 and LEU83. Although the original atomic grid did not estimate strong interactions in these regions, the MSMD-based GFEs exhibited strong, well-defined distributions. This indicates enhanced detection of critical protein–ligand hydrogen bonding patterns.

**Figure 5:**
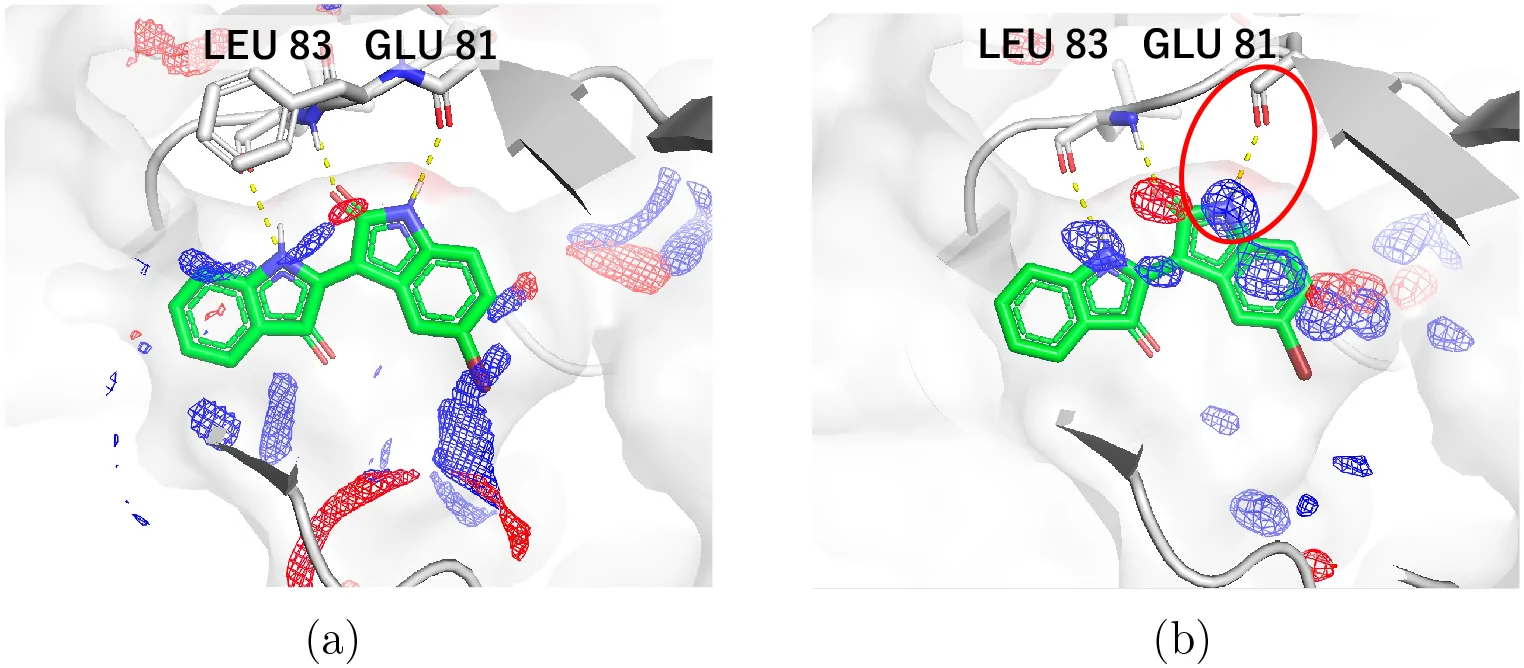
CDK2 protein structure (gray, PDB ID: 1H00) and binding pose of an inhibitor (green, PDB ID: 2BHE), comparing (a) interactions calculated by the original scoring function and (b) interactions calculated by the MSMD-based GFEs. The free energy distributions represent the hydrogen bond donor nitrogen atom (blue) and hydrogen bond acceptor oxygen atom (red). Dashed yellow lines indicate hydrogen bonds.

Analysis of the CDK2 structure during the simulation using formamide as a probe showed that the probe bound to the LEU83 and GLU81 residues, as illustrated in Figure 6. The ability to capture binding poses that simultaneously form two hydrogen bonds represents a major advantage over single-atom grids, which cannot account for such multipoint interactions. When the XS_type is classified as N_D, the nitrogen atom does not function as a hydrogen-bond acceptor. This classification suggests that the lone pair on the nitrogen is delocalized through conjugation, which typically only occurs in a limited set of chemical environments. Representative cases include aromatic systems observed in cocrystal structures or amide groups, such as those present in formamide. In this molecular context, the electronic structure of an atom is significantly affected by its surrounding chemical environment. Consequently, the ability of MSMD to capture these context-dependent electronic characteristics, rather than treating each atom as an isolated entity, is a major advantage of MSMD-based GFE.

**Figure 6:**
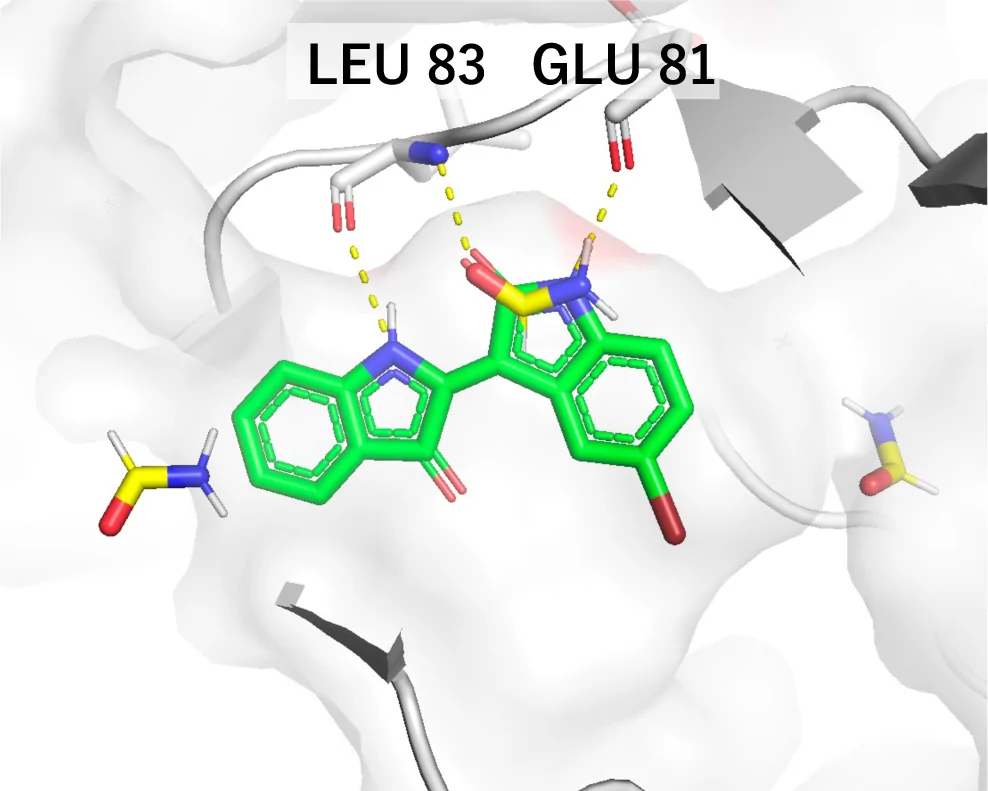
CDK2 protein structure (gray), formamide probe (yellow) in MSMD simulation, and binding pose of an inhibitor (green, PDB ID: 2BHE). Dashed yellow lines indicate hydrogen bonds.

### Analysis of Accuracy Deterioration in AmpC

The application of the proposed method resulted in a deterioration of accuracy across all evaluation metrics for AmpC. Therefore, we analyzed the complex structure of the AmpC protein with its cocrystallized ligand, as shown in Figure 7. In AmpC, hydrogen bonds are formed between the ligand and the residues ASN152 and ALA318. However, as shown in Figure 7b, hydrogen bonds with protein residues in the central region of the binding site were not sufficiently reproduced.

**Figure 7:**
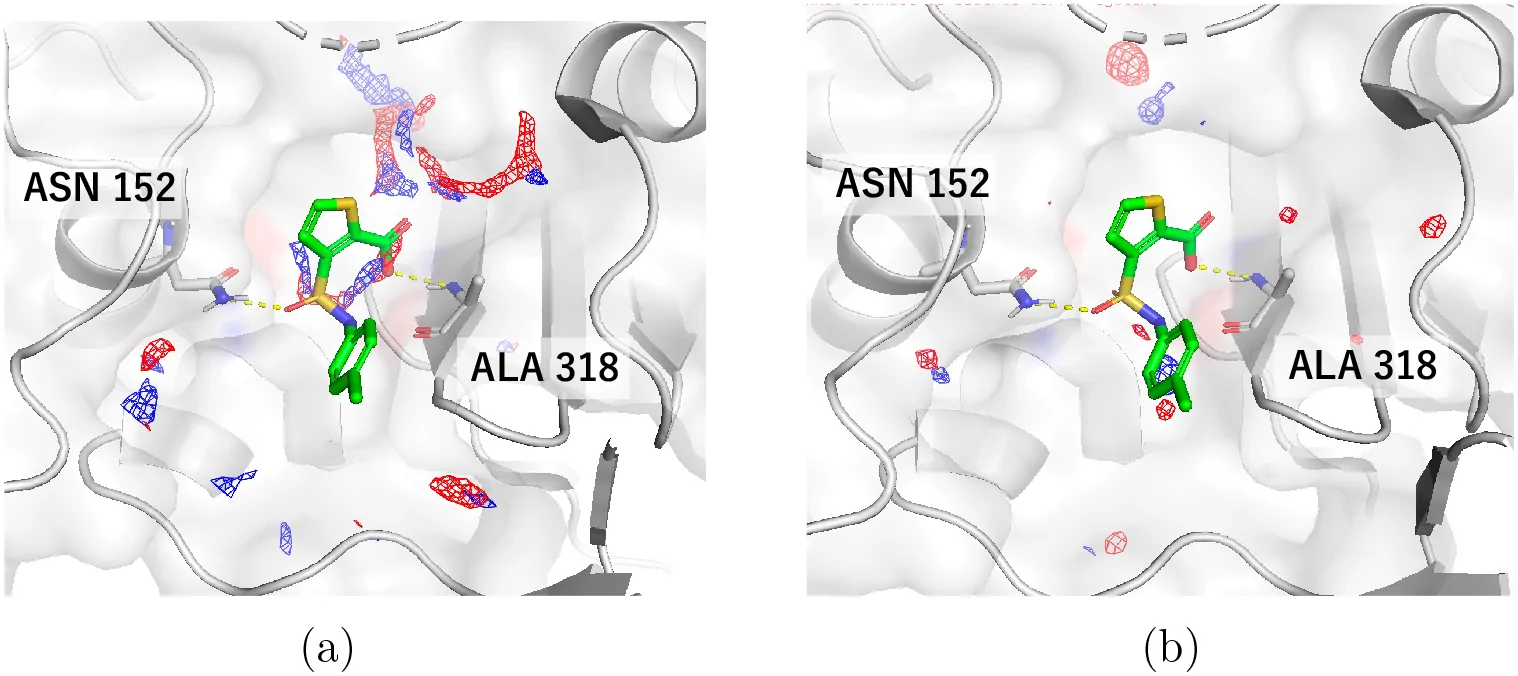
AmpC protein structure (gray) and binding pose of an inhibitor (green, PDB ID: 1L2S) displayed with (a) the original scoring function and (b) the MSMD-based GFE scores. The free energy distributions represent the hydrogen bond donor nitrogen atom (blue) and hydrogen bond acceptor oxygen atom (red). Dashed yellow lines indicate hydrogen bonds.

We further examined the docking pose of the decoy compound C38187871, which exhibited the highest docking score for AmpC in GFE-based VS, as shown in Figure 8. Although C38187871 adopted a docking pose similar to that of the cocrystallized ligand with the original scoring function, it was guided toward the upper region of the binding site when GFE was used. This observation was attributed to the fact that the distribution of the acceptor oxygen atoms, which was scored favorably by the original scoring function, was not emphasized in the GFE. Further examination of the cocrystallized ligand revealed that it adopted a carboxyl-like structure. Therefore, future studies can improve the accuracy by applying probes that consider charged groups.

**Figure 8:**
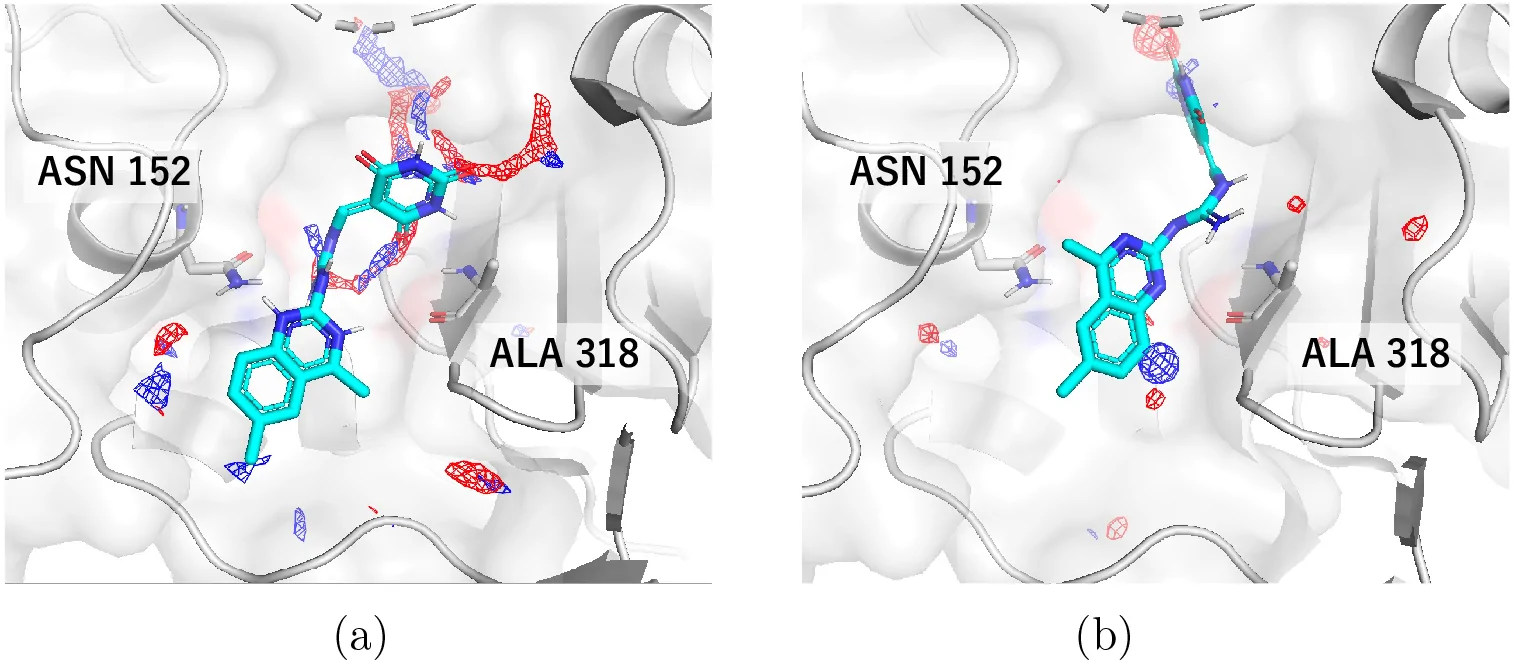
AmpC protein structure (gray, PDB ID: 1L2S) and the docking pose of the decoy compound C38187871 (cyan), showing the results obtained using (a) the original scoring function and (b) the MSMD-based GFEs. The free energy distributions represent the hydrogen bond donor nitrogen atom (blue) and hydrogen bond acceptor oxygen atom (red).

### The Use of Multiple Probes Improved Accuracy

We compared two conditions to validate our hypothesis that the use of multiple probes improves the prediction accuracy: a four-probe case, in which all four probe molecules (acetaldehyde, acetonitrile, formamide, and ethanol) were used, and an ethanol-only case. For the ethanol-only case, we mapped the XS_types according to our methodology, corresponding O_DA to the O1 atom and C_P to the C2 atom of ethanol, as shown in Figure 2. The four-probe case achieved a higher VS accuracy than the ethanol-only case, as shown in Table 8. For FGFr1 and PDE5A, the ethanol-only case performed worse than the baseline without GFEs, whereas the four-probe case did not exhibit a similar degradation. Comprehensive analysis of cases with reduced accuracy revealed that the oxygen atom capable of acting as both a hydrogen bond donor and acceptor (O_DA) was absent from the crystallized ligand of PDE5A. In contrast, O_DA was present in the crystallized ligand of FGFr1, yet did not contribute to protein binding, as shown in Figure 9. The MSMD-based method tended to produce higher scores with more pronounced distributions at specific locations than the original AutoDock Vina scoring function. Therefore, the scores may be excessively inflated when the decoy compounds contain O_DA atoms, potentially leading to decreased accuracy. These results demonstrate that increasing the variety of probe molecules improves the VS performance.

**Table 8:** Comparison of EF_1%_ Values for Each Protein^a^

		With GFEs	
Target	Without GFEs	Ethanol only	4 Probes
AmpC	6.26	**6.36**	1.29
BRD4	1.32	**1.82**	**3.70**
CDK2	8.61	**8.92**	**13.78**
DHFR	1.11	**1.48**	**1.82**
FGFr1	12.07	10.05	**15.07**
FXa	13.32	11.82	12.27
Gal-3	0.00	0.00	0.00
HIVpr	3.53	2.64	2.65
PDE5A	13.65	13.04	**15.68**
Average	6.65	6.24	**7.36**

a MSMD-based GFEs are subdivided into ethanol-only and four probes. Values showing better accuracy than those obtained without GFE are shown in bold.

**Figure 9:**
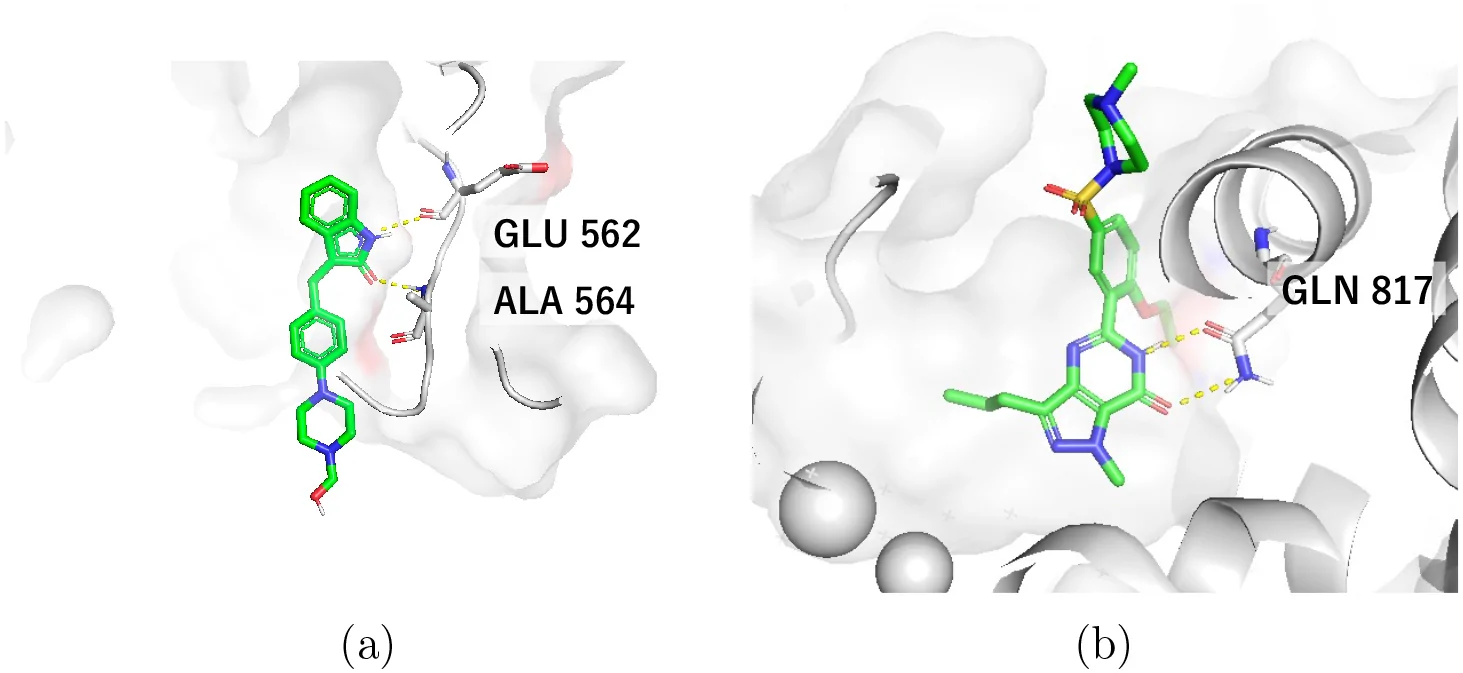
(a) The FGFr1 protein structure (gray) and binding pose of an inhibitor (green, PDB ID: 1AGW). (b) The PDE5A protein structure (gray) and binding pose of an inhibitor (green, PDB ID: 1UDT). Dashed yellow lines indicate hydrogen bonds.

### MSMD Is Computationally Practical for Large-Scale VS

In this study, we aimed to improve the accuracy of large-scale VS.^47^ As shown in Table 7, the computational cost of AutoDock Vina is approximately 2 CPU core minutes per compound. Consequently, docking calculations for 10 million compounds require approximately 1,700 node hours on the TSUBAME4.0 computing nodes (192 CPU cores per node), which corresponds to running 34 of the 240 available nodes for 50 h; this scale is well within our computational reach. In contrast, the MSMD simulations in our proposed method require only 200 node hours. As shown in the Computing Environment Section, these simulations require approximately one-eighth of the cost of AutoDock Vina docking calculations. Therefore, our method may be a useful approach for improving compound selection accuracy in such cases. However, the probe molecules selected in the proposed method were limited to neutral species, and although incorporating ionized probes to capture charged interactions would likely improve VS accuracy, doing so would inevitably increase computational cost; this trade-off warrants careful consideration in future work.

## Conclusions

In this study, we proposed a novel method for improving the accuracy of VS by integrating MSMD simulations with docking calculations. Using four types of probes (acetaldehyde, acetonitrile, formamide, and ethanol), we calculated the GFEs for multiple XS_types from MSMD simulations and combined them with the atomic grids provided by the AutoDock Vina scoring function, thereby incorporating protein flexibility and solvation effects into the scoring. Specifically, we applied GFEs to five XS_types, including oxygen and nitrogen atoms capable of forming hydrogen bonds and polar carbons.

Evaluation across nine target proteins demonstrated that our proposed method improved VS accuracy, with the average EF_1%_ increasing from 6.65 to 7.36 and the average ROC-AUC increasing from 0.659 to 0.671. Our method is advantageous compared to the previous approach by Arcon et al.; it avoids simplification of spherical regions, thereby better representing complex binding site geometries and utilizing multiple probe molecules to capture diverse interactions. Analysis of CDK2 revealed that the formamide probe could capture poses that simultaneously satisfied two hydrogen bonds. This major advantage cannot be achieved with single-atom atomic grids.

In the future, expanding the variety of probe molecules—including purely hydrophobic probes to better account for C_H-dominated interactions—is expected to capture more diverse interactions and improve the accuracy for a broader range of target proteins; our preliminary tests indicated that such probes require careful control of probe size and of the allowed protein flexibility. In this study, we applied GFE correction to five XS_types using four probe types, adopting an approach that modifies only a portion of the docking score rather than the entire score. A further extension would be to replace the entire docking score with GFE-based scoring. In that context, simultaneous incorporation of multiple probes may also be a viable option alongside the use of separately introduced single probes, as it would account for probe competition and is expected to more faithfully reproduce competitive binding among diverse functional groups.

## Supplementary Material



## Data Availability

Compounds and protein structures were obtained from the Directory of Useful Decoys Enhanced (DUD-E). The structures not available in DUD-E were obtained from the Supporting Information of Arcon et al.^21^ We used LigPrep (in the Schrödinger suite, version 2018-1) to generate stereoisomers and ionization states. The AutoDock Vina docking tool (version 1.2.0) was used to evaluate the effectiveness of the proposed approach. The open-source program PyMOL was used for visualization.

## Notes

Figures S1–S9: ROC curves (without GFEs, with GFEs) for each target (PDF)

## Abbreviations

AmpC: AmpC β-lactamase
AUC: area under the ROC curve
BRD4: bromodomain-containing protein 4
CDK2: cyclin-dependent kinase 2
CPU: central processing unit
DHFR: dihydrofolate reductase
EF: enrichment factor
FGFr1: fibroblast growth factor receptor 1
FXa: factor Xa
Gal-3: galectin-3
GFE: grid free energy
HIVpr: human immunodeficiency virus type 1 protease
MD: molecular dynamics
MSMD: mixed-solvent molecular dynamics
OC: overlap coefficient
PDB: Protein Data Bank
PDE5A: phosphodiesterase 5A
PMAP: probability distribution map
ROC: receiver operating characteristic
VS: virtual screening
