# Supplementary material for: Probe-Atom Distributions Obtained from Mixed-Solvent Molecular Dynamics Improve the Scoring of Docking Calculations
Source: J Chem Inf Model. 2026 Aug 31;66(17):10971–81. doi: 10.1021/acs.jcim.6c00947 (PMC13580117; doi:10.1021/acs.jcim.6c00947)
Supplement: Supplementary file 1 [file ci6c00947_si_001.pdf]

Supporting Information: Probe-Atom Distributions Obtained from Mixed-Solvent Molecular Dynamics Improve the Scoring of Docking Calculations

Kaho Akaki,[†] Keisuke Yanagisawa,[†,‡] and Yutaka Akiyama[*,†,‡]

†*Department of Computer Science, School of Computing, Institute of Science Tokyo, Meguro-ku, Tokyo 152-8550, Japan*

‡*Middle Molecule IT-based Drug Discovery Laboratory, Institute of Science Tokyo, Meguro-ku, Tokyo 152-8550, Japan*

E-mail: akiyama@comp.isct.ac.jp

Supporting Figures

GFE denotes the grid free energy derived from mixed-solvent molecular dynamics (MSMD). In each figure, w/o GFEs and w/ GFEs indicate the results obtained without and with the proposed GFE-based modification of the scoring function, respectively.

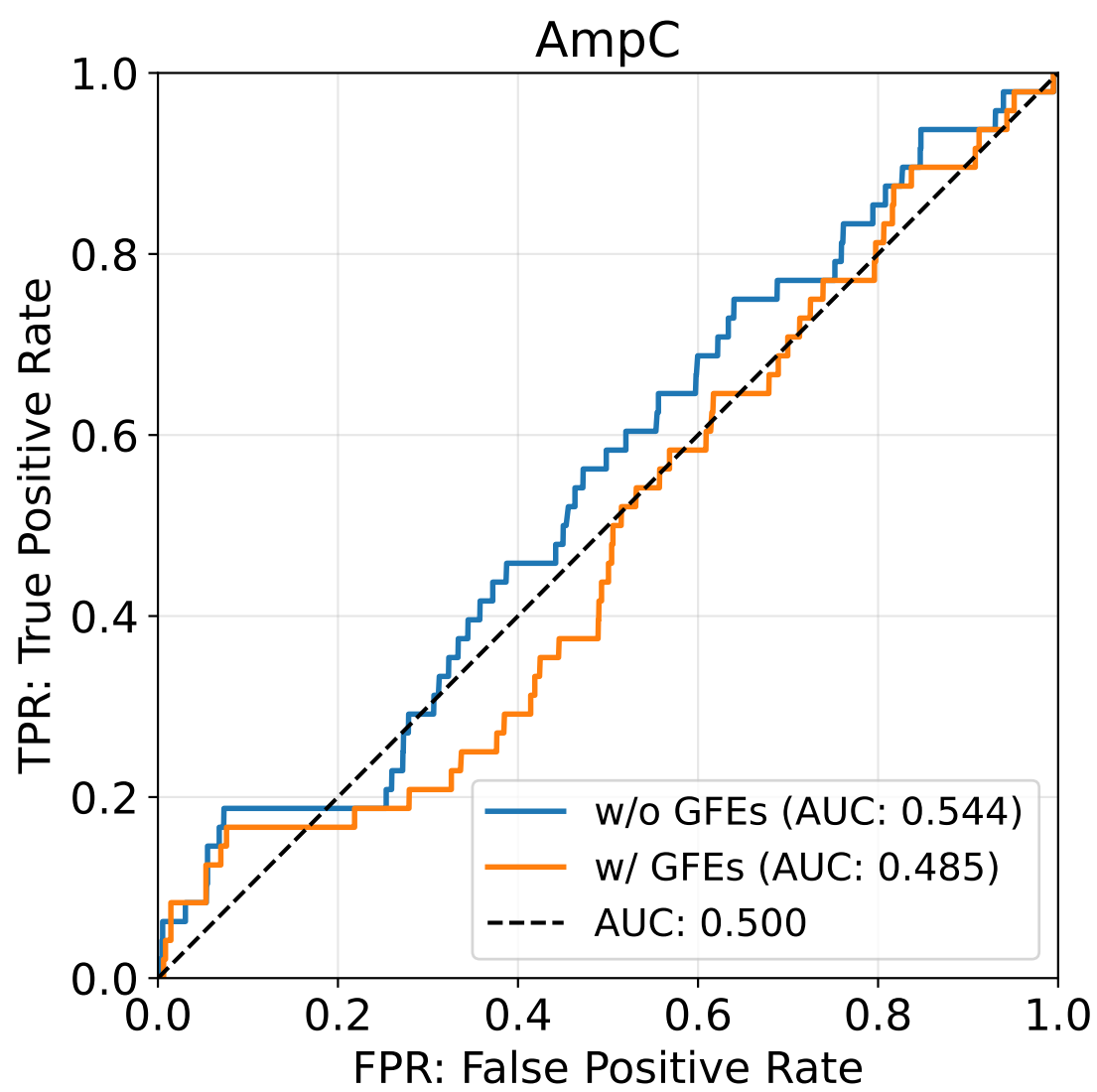

Figure S1: ROC curves for AutoDock Vina for the target AmpC.

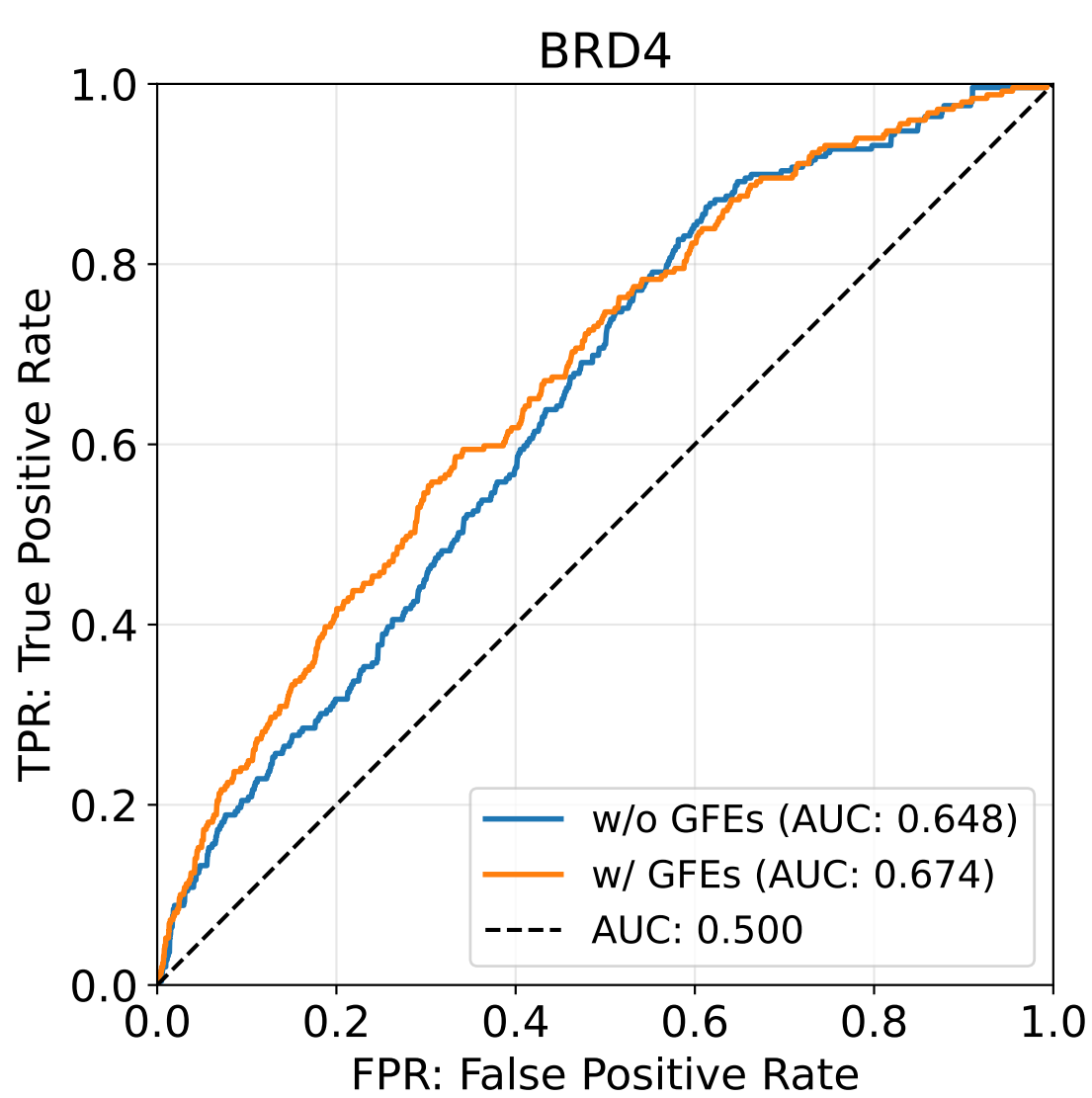

Figure S2: ROC curves for AutoDock Vina for the target BRD4.

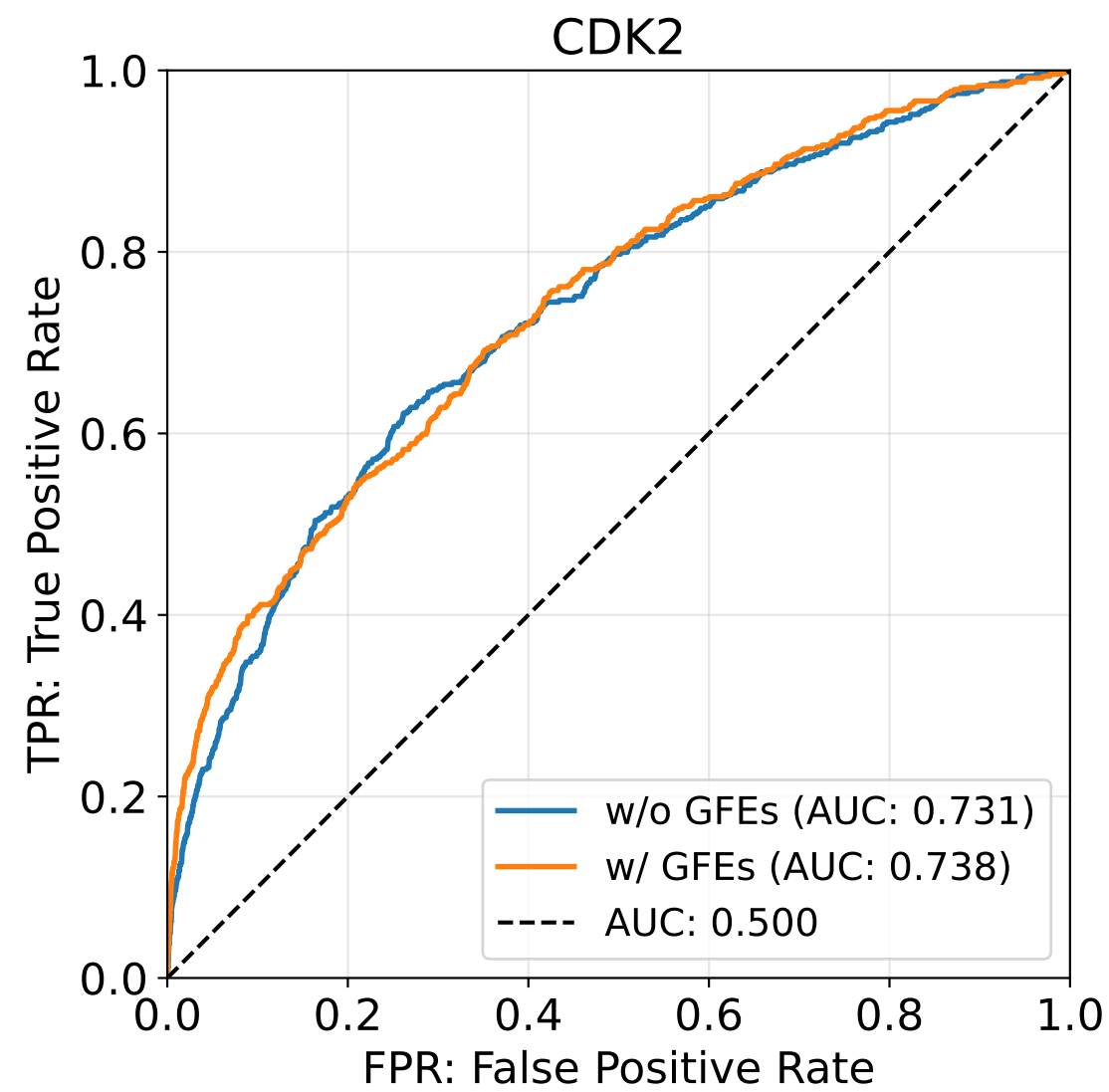

Figure S3: ROC curves for AutoDock Vina for the target CDK2.

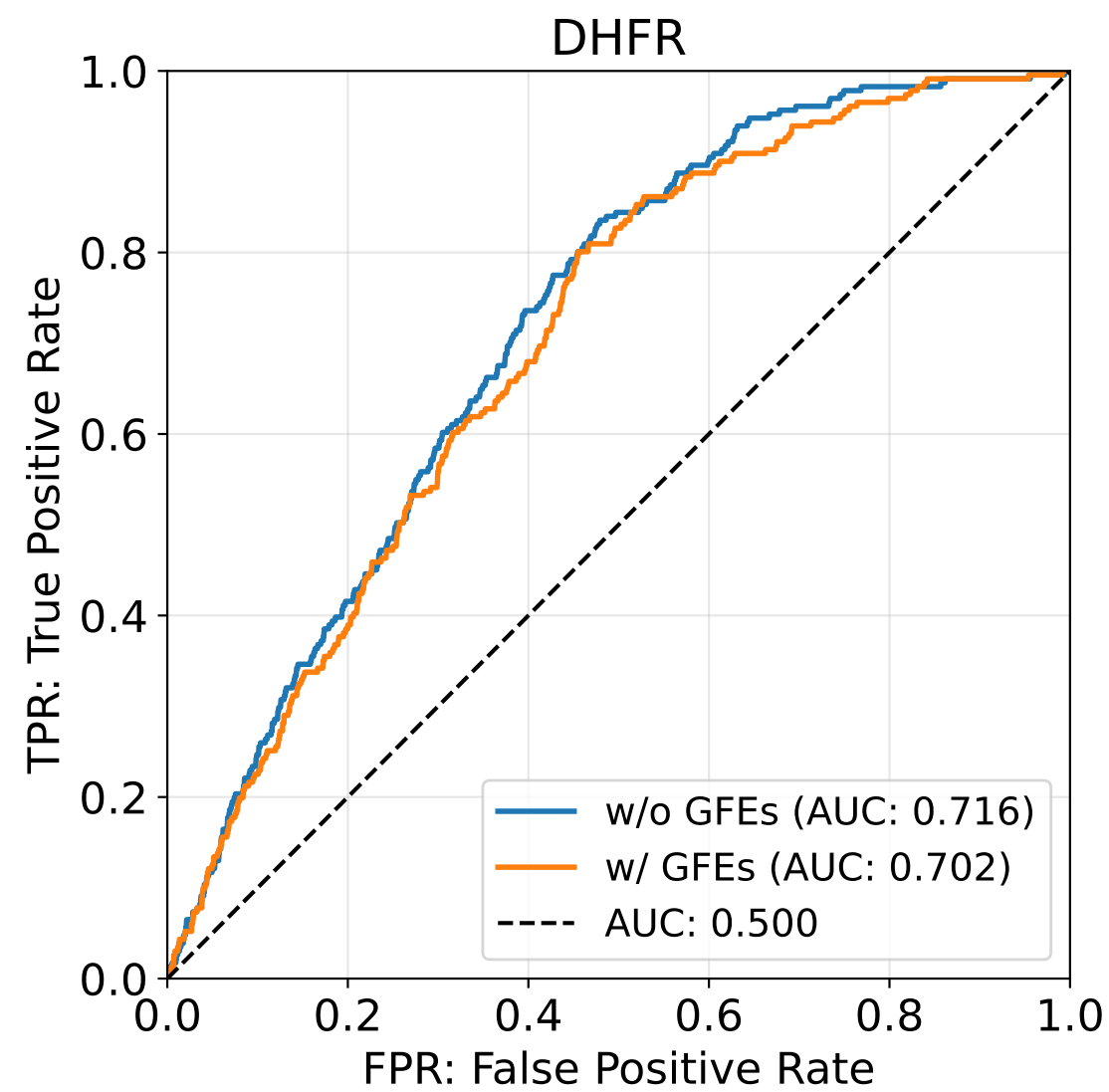

Figure S4: ROC curves for AutoDock Vina for the target DHFR.

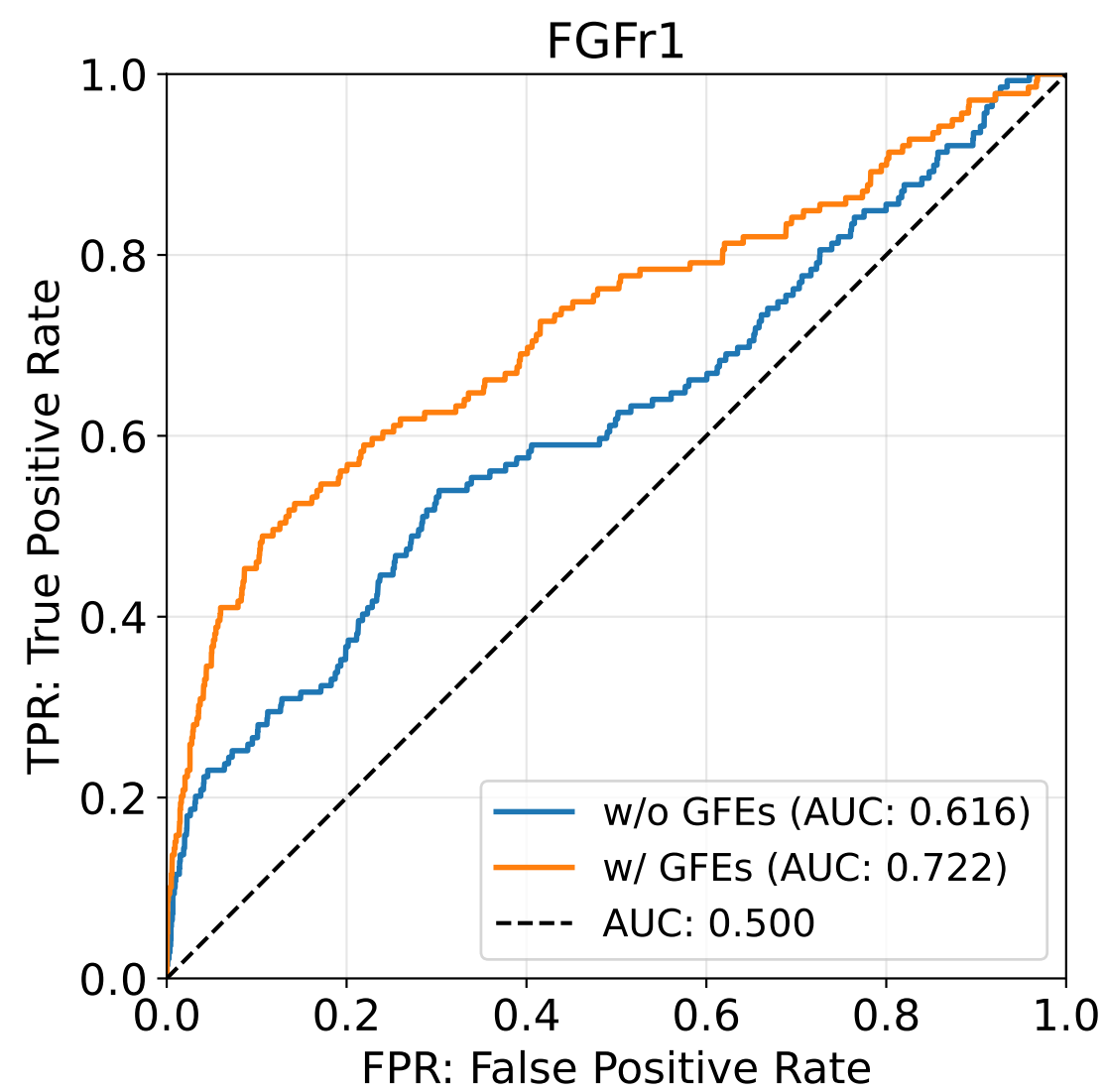

Figure S5: ROC curves for AutoDock Vina for the target FGFr1.

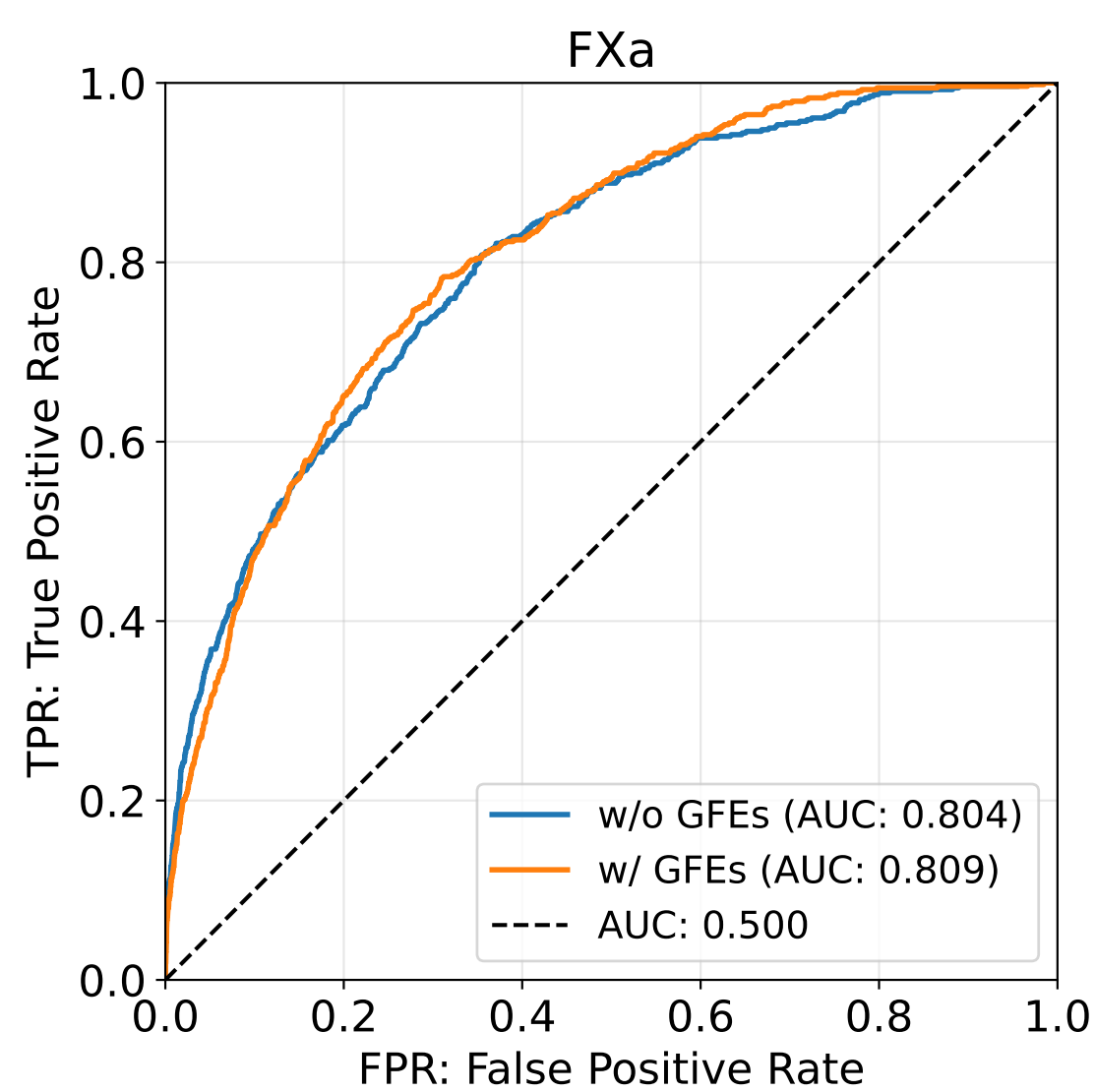

Figure S6: ROC curves for AutoDock Vina for the target FXa.

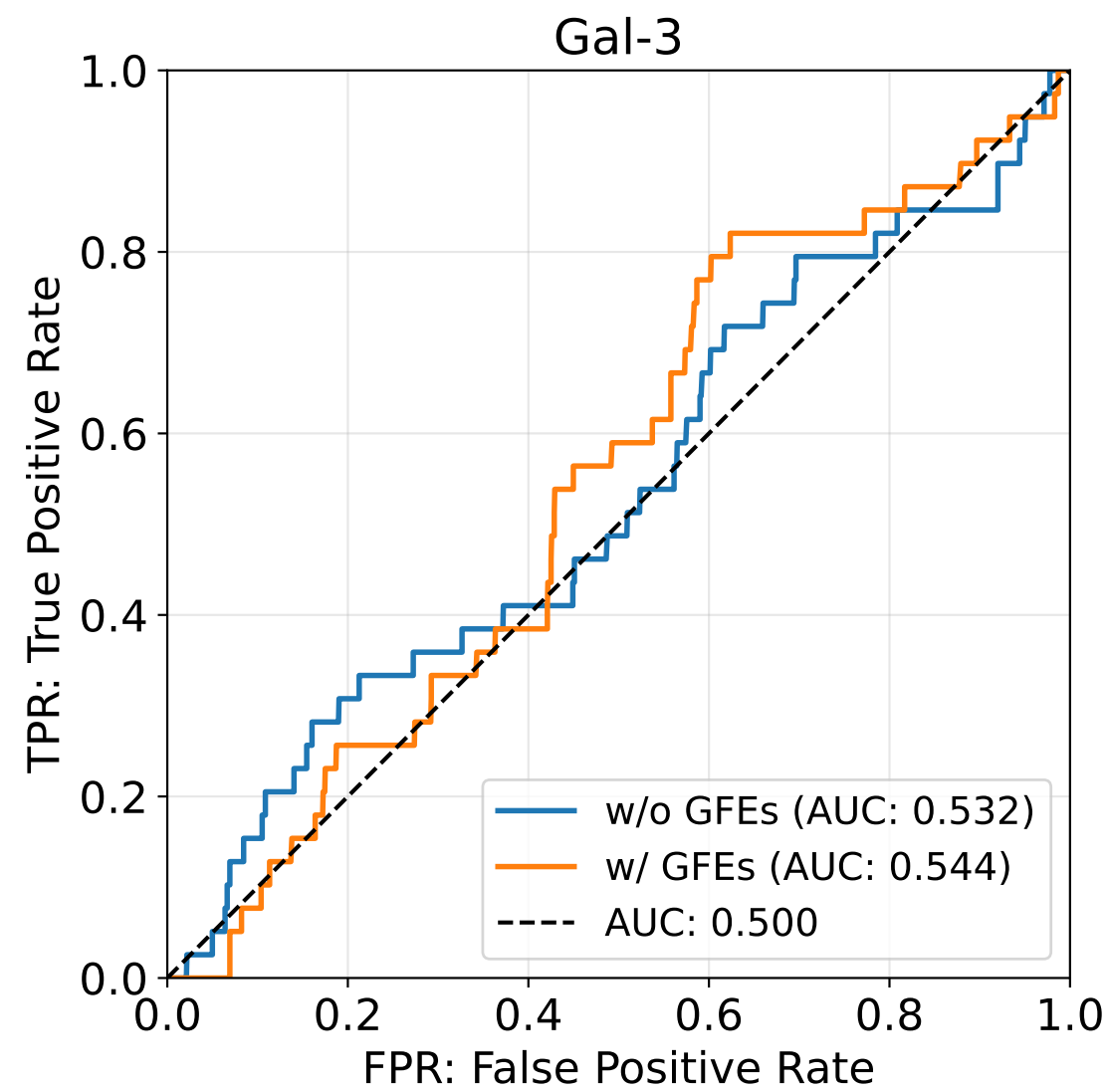

Figure S7: ROC curves for AutoDock Vina for the target Gal-3.

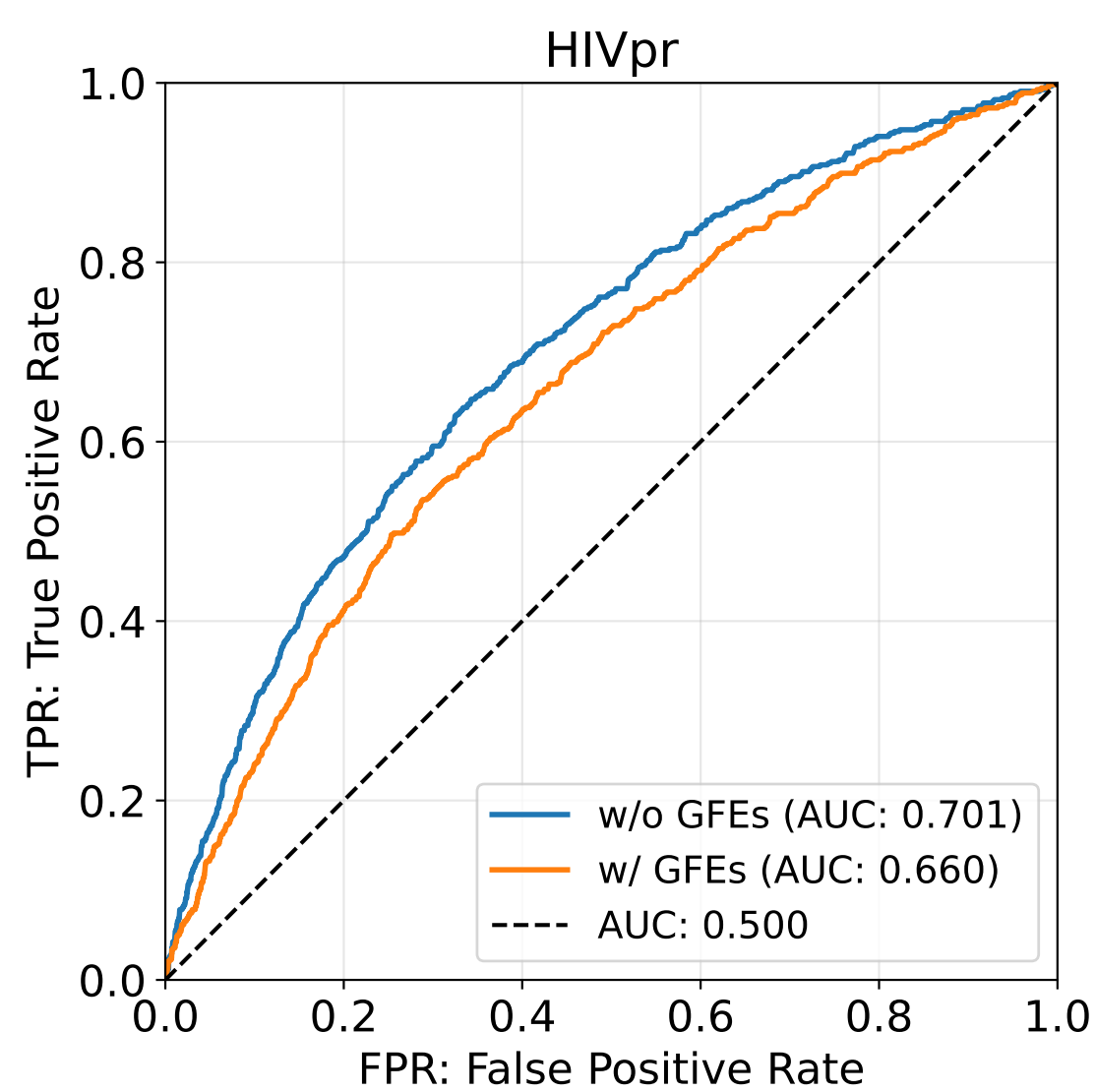

Figure S8: ROC curves for AutoDock Vina for the target HIVpr.

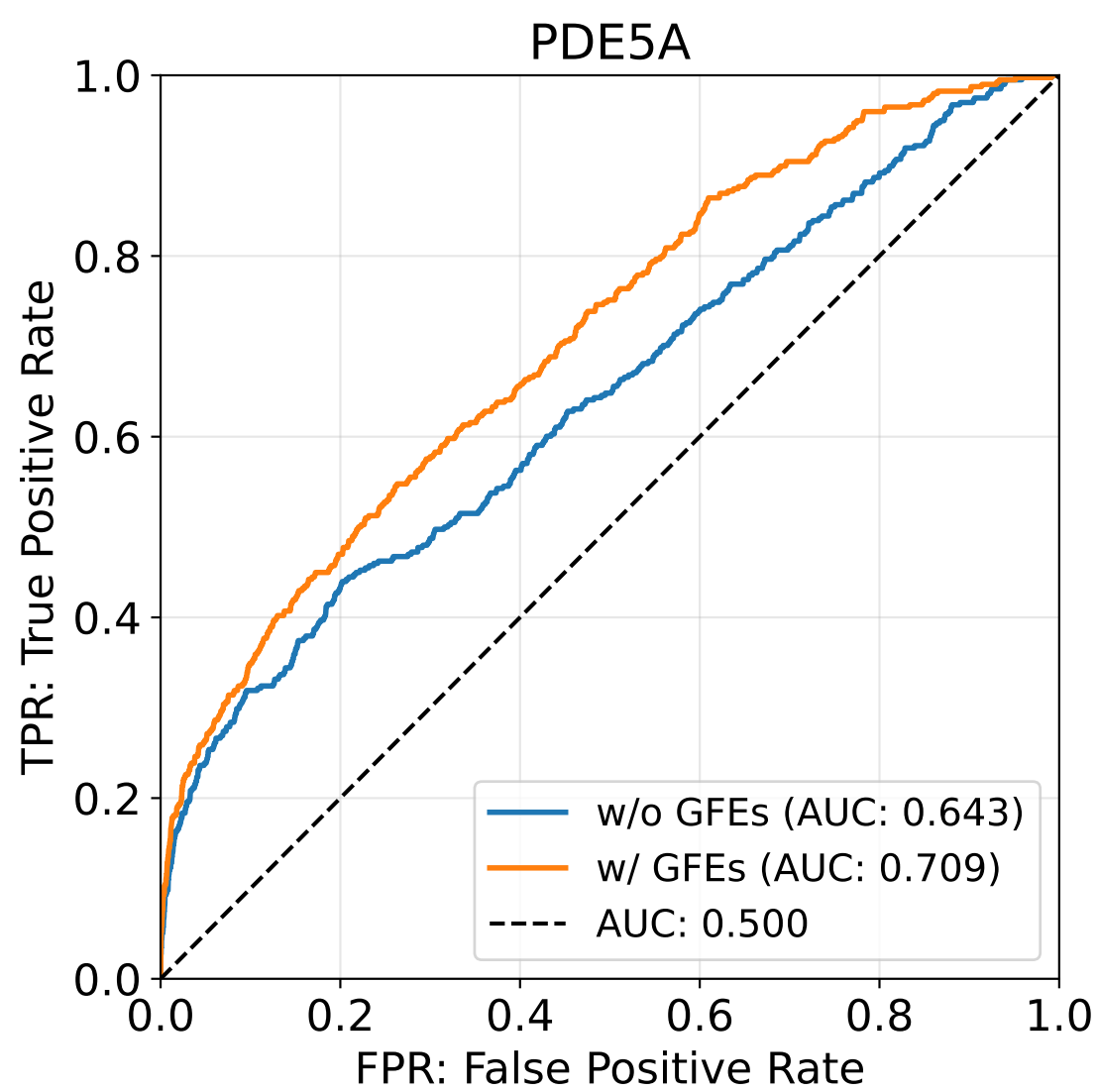

Figure S9: ROC curves for AutoDock Vina for the target PDE5A.
